# An intrathyroidal cystic parathyroid adenoma localised with [^18^F]fluorocholine PET/CT in pregnancy

**DOI:** 10.1186/s41824-026-00311-w

**Published:** 2026-06-29

**Authors:** Sameer K. Dave, Claire Mok, Stan Sidhu, John Turchini, Margaret Layton, Weng Sam, David Medveczky, Jeremy Hoang

**Affiliations:** 1 Royal North Shore Hospital, Sydney, NSW Australia; 2 University of Sydney, Sydney, NSW Australia; 3 Douglass Hanly Moir Pathology, Sydney, NSW Australia; 4 Macquarie University, Sydney, NSW Australia; 5 Gosford Hospital, Gosford, NSW Australia; 6 Private Practice, North Gosford, NSW, Australia

**Keywords:** Primary hyperparathyroidism, Pregnancy, [^18^F]fluorocholine PET/CT, Intrathyroidal parathyroid adenoma, Cystic parathyroid adenoma, Low-dose imaging, 4D CT, Ultrasound, Parathyroid localisation

## Abstract

Primary hyperparathyroidism during pregnancy is rare but associated with substantial maternal and foetal morbidity, necessitating timely diagnosis and intervention. Intrathyroidal parathyroid adenomas account for as few as 0.7% of parathyroid adenomas, while cystic parathyroid adenomas represent an even smaller subset. The coexistence of both is exceedingly uncommon, with only nine cases reported and none previously described in pregnancy or using [^18^F]fluorocholine PET/CT for localisation. We describe a 37-year-old pregnant woman with PTH-dependent hypercalcaemia which persisted following initial parathyroidectomy. A left thyroid lesion with mixed cystic and peripheral enhancing components was detected on ultrasound and multiphase IV contrast CT (4D CT) but remained indeterminate. To minimise foetal radiation exposure, an ultra-low dose protocol [^18^F]fluorocholine PET/CT was performed at 16 weeks’ gestation, demonstrating peripheral tracer uptake consistent with a hyperfunctioning intrathyroidal cystic parathyroid adenoma. Ultrasound-guided sampling confirmed markedly elevated cyst fluid PTH. Definitive management with left hemithyroidectomy at 23 weeks’ gestation resulted in normalisation of biochemistry and an uncomplicated term delivery. This first reported case of intrathyroidal cystic parathyroid adenoma in pregnancy localised with [^18^F]fluorocholine PET/CT highlights the diagnostic challenges of primary hyperparathyroidism in pregnancy and supports carefully justified, dose-optimised functional imaging when conventional modalities are inconclusive and surgical decision-making depends on accurate localisation.

## Introduction

Primary hyperparathyroidism (PHPT) is the third most common endocrine disorder, with a higher prevalence in women and a peak incidence in the postmenopausal period, though it can also occur during pregnancy (Som and Stroup 2011; Ali et al. 2021; Petranović Ovčariček et al. 2021). PHPT in pregnancy poses significant risks to both mother and foetus, including hypercalcaemic crisis, nephrolithiasis, pancreatitis, miscarriage, preterm delivery, intrauterine growth restriction and neonatal hypocalcaemia, with maternal complication rates reported as high as 67% and foetal loss rates as high as 80% in severe, uncontrolled cases (Som and Stroup 2011; Ali et al. 2021; Petranović Ovčariček et al. 2021).

Intrathyroidal parathyroid adenomas (IPA) are an uncommon cause of PHPT, accounting for 0.7–6% of cases (Gowrishankar et al. 2023). Cystic parathyroid adenomas are rarer still, accounting for only 0.08–3.41% of parathyroid lesions, of which 10–15% can be functional (Taguchi et al. 2016). The combination of the two pathologies, intrathyroidal cystic parathyroid adenomas (ICPA) are exceedingly rare, with only nine cases reported to date in the literature (Gowrishankar et al. 2023; Grimley and Davies 1977; Chen et al. 2019; Hansa et al. 2023; Daoud and Ronen 2023; Sadacharan et al. 2015; Scheidt et al. 2020). The pathogenesis of cystic change in parathyroid adenomas may relate to cystic degeneration or intra-cystic haemorrhage, the latter being a recognised but uncommon presentation (Taguchi et al. 2016). In current literature, none of these previously reported cases occurred in pregnancy, and none involved localisation with [^18^F]fluorocholine PET/CT.

We present the first reported case of a cystic intrathyroidal parathyroid adenoma diagnosed during pregnancy and localised pre-operatively with [^18^F]fluorocholine PET/CT. This case highlights the unique diagnostic and management challenges of PHPT in pregnancy, the rare occurrence of ICPA and the careful balance between diagnostic accuracy and foetal safety in the use of functional imaging.

## Case

A 37-year-old woman with a background of medullary sponge kidney with nephrocalcinosis and nephrolithiasis, Hashimoto’s thyroiditis and metabolic associated fatty liver disease presented with hypercalcaemia. There was no family history of calcium disorders nor a history of Lithium use. Investigations revealed a serum calcium of 2.9 mmol/L corrected (normal range 2.15–2.55), PTH 25.7 pmol/L (normal range 1.6–6.9), eGFR > 90 ml/min/1.73 m^2^ and 25(OH)vitamin D level 82 nmol/L (normal range 50–140). The 24-hour urine calcium was 8.0 mmol and creatinine 11.1 mmol. The calculated urinary fractional excretion of calcium was 1.5%, suggestive of primary hyperparathyroidism.

**Fig. 1:**
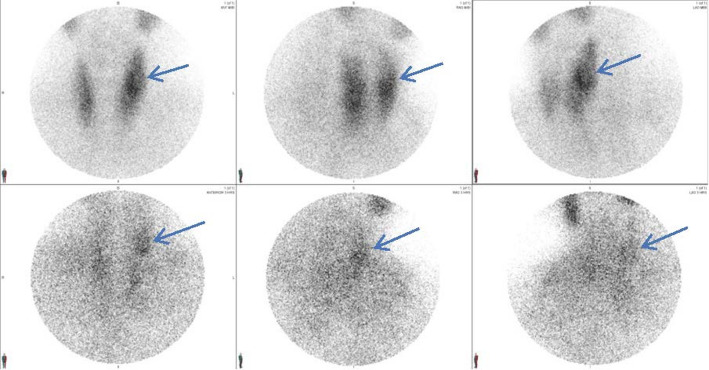
20-minute (top) and 3-hour (bottom) delayed planar [99mTc]sestamibi images showing subtly increased focal uptake in the region of the left mid-upper thyroid (blue arrows)

**Fig. 2:**
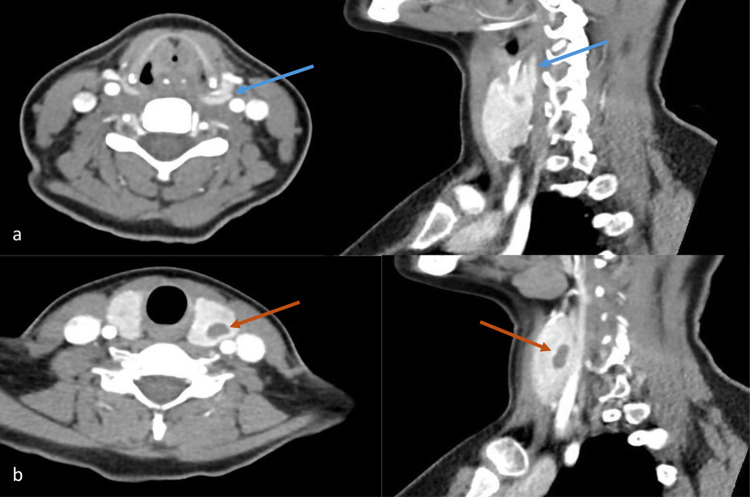
(**a**) Axial and sagittal arterial phase images of 4D CT showing an enhancing lesion posterior to the left upper thyroid pole (blue arrows) and (**b**) the same study showing a hypodense left thyroid lobe lesion (orange arrows)

On [99mTc]sestamibi parathyroid imaging, she was found to have tracer accumulation in the region of the left mid-upper thyroid lobe (Fig. 1). Unfortunately, the study was performed at an external centre, and the source images of the SPECT/CT were unavailable for review. Pre-operative 4D CT demonstrated an adenoma in this region as well as a presumed hypodense left thyroid nodule (Fig. 2).

**Fig. 3:**
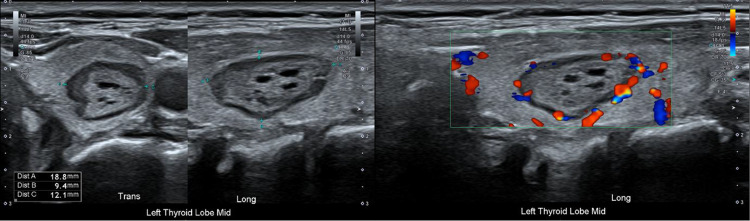
Left – Ultrasound showing an atypical left thyroid nodule with hypoechoic rim and heterogeneous central component with cystic foci. Right – Doppler image showing peripheral hyperaemia

**Fig. 4:**
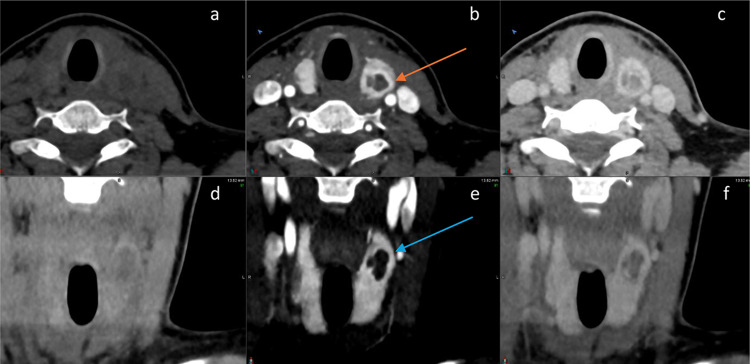
Axial and coronal images of 4D CT showing a left thyroid lesion in pre-contrast (**a**/**d**), arterial (**b**/**e**) and delayed phases (**c**/**f**). There is peripheral arterial hyper-enhancement (orange arrow) which is relatively hypodense on the pre-contrast and delayed phases. The central component does not enhance (blue arrow)

She proceeded to a four-gland surgical exploration, which found a left superior parathyroid adenoma. This was fully resected and confirmed on histopathology, weighing 661 mg. Despite this, post-operatively, the patient had a persistently elevated plasma PTH of 20 pmol/L and persistently elevated corrected calcium of 2.77 mmol/L. A post-operative ultrasound (Fig. 3) and 4D CT (Fig. 4) redemonstrated the left thyroid nodule, now with more atypical features including cystic change, peripheral hyperaemia and arterial hyper-enhancement.

**Fig. 5:**
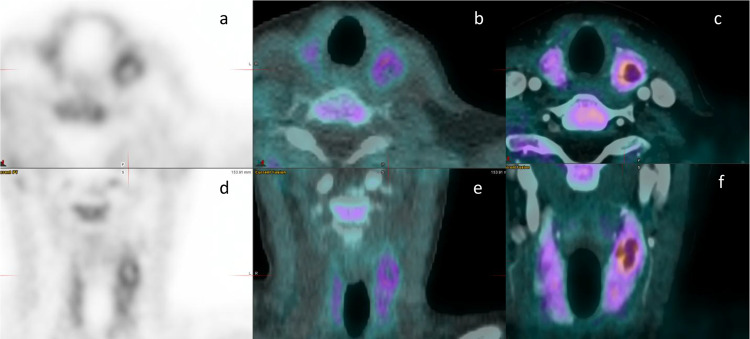
Axial and coronal images of [^18^F]fluorocholine PET (**a**/**d**), PET fused with low dose CT (**b**/**e**) and PET fused with arterial phase 4D CT (**c**/**f**). The PET/CT was performed on a Siemens Biograph Vision Quadra total-body digital camera. The periphery of the nodule shows increased [^18^F]fluorocholine uptake (SUVmax 4.0 compared to blood pool SUVmax 0.4), consistent with a parathyroid adenoma

The patient was referred for an [^18^F]fluorocholine PET/CT with the aim to improve localisation of suspected hyperfunctioning parathyroid tissue. However, she became pregnant in the interim which required delay of imaging due to radiation risks to the foetus. The PET scan was performed at 16 weeks’ gestation using an ultra-low dose protocol. The injected activity was 46 MBq (0.68 MBq/kg) compared to a standard injected activity of 120 MBq at our institution and EANM recommended activity of 100–300 MBq or approximately 3 MBq/kg (Petranović Ovčariček et al. 2021). A longer PET acquisition time of 15 minutes was used, compared with a standard 5 minute acquisition on the high-sensitivity digital PET/CT scanner (Siemens Biograph Vision Quadra). Ultra-low dose CT parameters which included tin filtration were also utilised. The PET/CT scan demonstrated peripheral tracer uptake in the left thyroid lesion corresponding to the peripheral hyper-enhancement on CT, and a photopaenic central component corresponding to the cystic portion of the lesion (Fig. 5). These findings confirmed what was initially thought to be a complex thyroid nodule was in fact a second separate parathyroid adenoma, completely intrathyroidal and distinct from the previously resected parathyroid adenoma. This was further supported by findings on ultrasound-guided sampling of this nodule, showing a fluid PTH of >318 pmol/L.

**Fig. 6:**
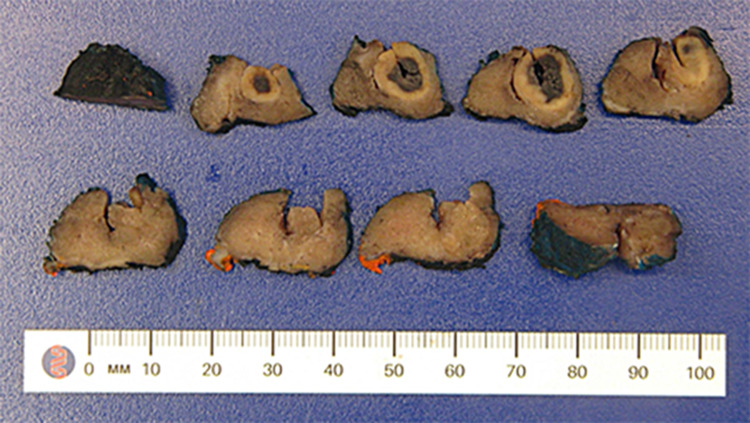
Macroscopic hemithyroidectomy showing the parathyroid lesion embedded within the thyroid parenchyma

**Fig. 7:**
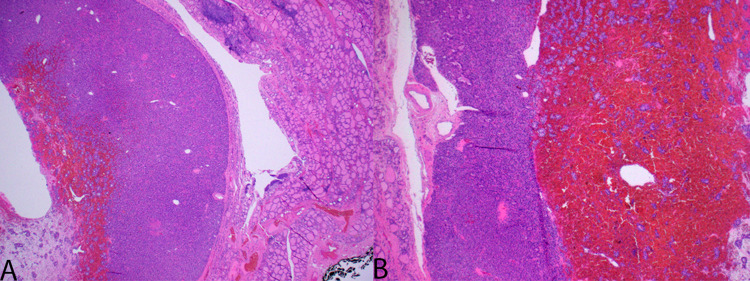
(**A**) Interface between parathyroid adenoma and thyroid (H&E, 40X). (**B**) Parathyroid adenoma demonstrating central cystic change and haemorrhage (H&E, 100X)

**Fig. 8:**
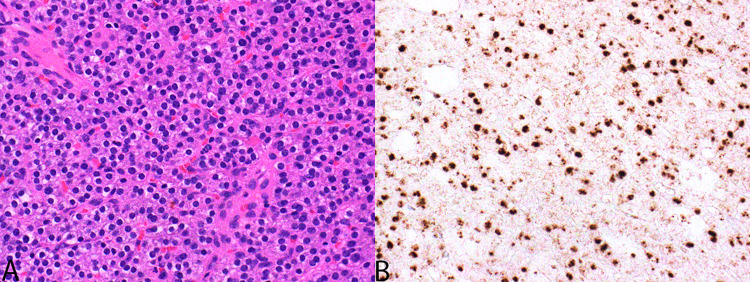
(**A**) Bland cytology of parathyroid cells (H&E, 400X). (**B**) Parafibromin immunohistochemistry showing retained nuclear staining (H&E, 400X)

She was managed with normal saline IV fluid to maintain a serum corrected calcium <2.75 mmol/L until a left hemithyroidectomy was performed at 23 weeks’ gestation. Histopathology demonstrated an 18 × 11×10 mm intrathyroidal parathyroid adenoma with a central cystic component secondary to haemorrhage (Figs. 6, 7 and 8). Post-operative recovery was uneventful, with normalisation of serum calcium and PTH levels. The patient completed her pregnancy without complications and delivered a healthy term infant. At 4 months post-operatively, both serum corrected calcium and PTH levels remained normal at 2.42 mmol/L and 3.7 pmol/L respectively, highly suggestive of cure. The patient declined genetic testing.

## Discussion

Primary hyperparathyroidism (PHPT) in pregnancy is rare but clinically significant due to its high complication rates for both mother and foetus. This case underscores the importance of a tailored, multidisciplinary approach to managing PHPT during pregnancy, particularly in rare presentations such as cystic intrathyroidal parathyroid adenoma. It shows that, with careful justification and adherence to radiation safety principles, [^18^F]fluorocholine PET/CT can be safely and effectively employed to achieve precise pre-operative localisation when conventional imaging is indeterminate. As the first documented case of [^18^F]fluorocholine PET/CT localisation of an intrathyroidal cystic parathyroid adenoma in pregnancy, it highlights both the diagnostic utility of advanced functional imaging and the need for flexible, evidence-informed decision-making in complex endocrine disorders encountered during pregnancy.

Given the significant risk associated with PHPT to the foetus and mother in pregnancy, definitive surgical management is generally recommended during the second trimester, when foetal organogenesis is complete and the risk of preterm labour is lower (Ali et al. 2021; Petranović Ovčariček et al. 2021). There are no formal, evidence-based guidelines for the diagnosis and management of PHPT in pregnancy, and management strategies must balance maternal and foetal safety while minimising diagnostic delay.

Diagnostic imaging in pregnancy requires careful consideration to limit foetal radiation exposure. Current European Association of Nuclear Medicine (EANM) guidelines recommend preferential use of non-ionising modalities such as high-resolution ultrasonography as first-line localisation for pregnant patients (Petranović Ovčariček et al. 2021). Functional imaging, while central to PHPT localisation in non-pregnant patients, is generally deferred in pregnancy unless it is essential for management, and in such situations, a careful risk benefit assessment is required. In Australia, the use of radiopharmaceuticals during pregnancy is governed by general radiation protection principles issued by the Australian Radiation Protection and Nuclear Safety Agency (ARPANSA); diagnostic nuclear medicine procedures may still be performed in pregnant patients when the expected clinical benefit outweighs the radiation risk.

[^18^F]fluorocholine PET/CT has emerged as a highly sensitive modality for localising parathyroid adenomas in PHPT, with previous studies demonstrating its superiority over conventional techniques since 2014 (Lezaic et al. 2014; Michaud et al. 2014). A more recent literature review further supports the use of [18F]fluorocholine PET/CT as a first line investigation, however notes that in some jurisdictions, this tracer is contraindicated for use in pregnancy (Noskovicova et al. 2024). While its use in pregnancy is not standard practice due to radiation exposure, its potential value in complex or equivocal cases is increasingly recognised in the non-pregnant population. In this case, first line [18F]fluorocholine PET/CT may have detected the two parathyroid adenomas and avoided the radiation exposure from [99mTc]sestamibi scintigraphy and 4D CT.

When functional imaging is performed in pregnancy, a low-dose protocol is recommended to minimise foetal radiation exposure. At our centre, an ultra-low dose PET protocol involves administration of a reduced [18F]fluorocholine activity of 40 MBq and scanning for 15 minutes on a high-sensitivity digital PET/CT scanner (Siemens Biograph Vision Quadra). This gives an estimated effective dose of 1.5 mSv to the mother. For comparison, our standard non-pregnancy protocol involves administration of an [18F]fluorocholine activity of 120 MBq and scanning for 5 minutes, giving an estimated effective dose of 4.4 mSv (DeGrado et al. 2000). We also employ an ultra-low dose CT protocol which uses tin filtration in the x-ray beam to remove the lower energy x-rays, giving a reduced effective dose of 0.2–0.3 mSv. For comparison, the typical effective dose from our CT component is 4–6 mSv. Hence, the total effective dose to the mother from the combined ultra-low dose protocol PET/CT is approximately 1.8 mSv. The total effective dose to the foetus estimated from the maternal dose is 1.0 mSv. This dose corresponds to a risk of induced cancer to the foetus approximately 100 times less than the accepted sporadic rate (Lowe 2020). Further, published data show no evidence of any increase in lethality (miscarriage or stillbirth), genetic damage, teratogenicity, growth impairment, mental retardation or sterility at doses less than 50 mSv (Lowe 2020).

This case also contributes to the known association between medullary sponge kidney and PHPT. The pathogenesis explaining this association is still unknown with some authors suggesting PHPT to be causative (Fabris et al. 2013). However, in many reports including our patient, the nephrocalcinosis preceded the diagnosis of PHPT by many years.

Our case underscores the need for cautious but flexible application of functional imaging techniques in pregnancy when the benefits clearly outweigh the risks. Given the lack of published guidelines on managing PHPT in pregnancy, each case requires individualised, multidisciplinary decision-making. Our case expands the existing literature by documenting the first pregnant patient with ICPA, the first pre-operative localisation of such a lesion in pregnancy using [^18^F]fluorocholine PET/CT, and the successful maternal–foetal outcomes following second-trimester surgical resection.

## Data Availability

Data sharing not applicable to this article as no datasets were generated or analysed during the current study.
